# Mechanical behaviour of healthy versus alkali-lesioned corneas by a porcine organ culture model

**DOI:** 10.1186/s12917-021-03050-1

**Published:** 2021-10-28

**Authors:** Chiara Giulia Fontanella, Emanuele Luigi Carniel, Livio Corain, Antonella Peruffo, Ilaria Iacopetti, Piero G. Pavan, Silvia Todros, Anna Perazzi

**Affiliations:** 1 Department of Industrial Engineering, University of Padova, via Venezia 1, 35131 Padova, Italy; 2 Centre for Mechanics of Biological Materials, University of Padova, via Marzolo 9, 35131 Padova, Italy; 3 Department of Management and Engineering, University of Padova, stradella S. Nicola 3, 36100 Vicenza, Italy; 4 Department of Comparative Biomedicine and Food Science, University of Padova, viale dell’Università 16, Legnaro, 35020 Padova, Italy; 5 Department of Animal Medicine, Production and Health, University of Padova, viale dell’Università 16, Legnaro, 35020 Padova, Italy

**Keywords:** Porcine cornea, Alkali-induced lesions, Riboflavin/UV-A corneal phototherapy, Biomechanical behaviour, Tensile tests, Statistical analysis

## Abstract

**Background:**

Cornea is a composite tissue exhibiting nonlinear and time-dependent mechanical properties. Corneal ulcers are one of the main pathologies that affect this tissue, disrupting its structural integrity and leading to impaired functions. In this study, uniaxial tensile and stress-relaxation tests are developed to evaluate stress-strain and time-dependent mechanical behaviour of porcine corneas.

**Results:**

The samples are split in two groups: some corneas are analysed in an unaltered state (healthy samples), while others are injured with alkaline solution to create an experimental ulcer (lesioned samples). Furthermore, within each group, corneas are examined in two conditions: few hours after the enucleation (fresh samples) or after 7 days in a specific culture medium for the tissue (cultured samples). Finally, another condition is added: corneas from all the groups undergo or not a cross-linking treatment. In both stress-strain and stress-relaxation tests, a weakening of the tissue is observed due to the imposed conditions (lesion, culture and treatment), represented by a lower stiffness and increased stress-relaxation.

**Conclusions:**

Alkali-induced corneal stromal melting determines changes in the mechanical response that can be related to a damage at microstructural level. The results of the present study represent the basis for the investigation of traditional and innovative corneal therapies.

**Supplementary Information:**

The online version contains supplementary material available at 10.1186/s12917-021-03050-1.

## Background

Ulcerative melting keratitis is a pathological condition of the cornea where stromal damage is probably caused by an imbalance between proteinases and proteinase inhibitors, which are components involved in corneal wound healing. This pathology alters the tissue microstructural configuration influencing its mechanical functionality. In recent years, research efforts, both in human and veterinary medicine, are directed to find alternative or complementary methods supporting the healing process of the corneal ulcerative diseases [1–3]. Great interest has been directed towards a method designed to strengthen the structure of the corneal stroma, working on the natural links between collagen fibres, as alternatives to graft and corneal transplantation [4].

The cornea has a nonlinear elastic, anisotropic and quasi-linear viscoelastic mechanical behaviour that depends on the cornea’s microstructure and composition [5]. Uniaxial tensile tests and inflation tests are the most used for ex vivo studies [5–11]. Tissue nonlinear elastic behaviour and stiffening with increasing intraocular pressure can be evaluated through inflation tests, while the stress-strain behaviour and the viscoelastic response in medium and long term can be evaluated in uniaxial tensile mode. Recently, optical coherence elastography [12] and ultrasound elastography [13] has been extensively used to characterize the mechanical properties of the cornea. Brillouin microscopy has been also applied for mapping corneal elastic modulus in three dimensions with a high spatial resolution [14]. Porcine corneas are frequently used to assess biomechanical properties as corneal models for other species. Indeed, despite some differences in corneal thickness, porcine cornea is largely accepted as a suitable model to estimate the mechanical properties of human or other animal cornea [7, 15, 16]. For example, the porcine and human cornea exhibit similar stress-strain behaviour under short- and long-term loading and react similarly to sustained loading, although the creep is higher in human than in porcine corneas [7].

In the recent years, there is an increasing interest in ophthalmology for riboflavin/UV-A corneal phototherapy, in particular in the treatment of corneal ulcers gaining success not only in human, but also in veterinary medicine [17, 18]. The riboflavin/UV-A corneal phototherapy is believed to increase stiffness and strength of the cornea. With this treatment, in the corneal stroma the riboflavin (vit. B12) and the UVA undergo a photochemical reaction with environmental oxygen and generate free oxygen radicals. This reaction is responsible for the formation of additional covalent bonds between the collagen fibrils in the stroma with the result of reinforcing the structure of corneal stroma influencing its biomechanical function [19]. The literature reports few data about the possible modification of mechanical behaviour of corneal tissue due the induced injury and also due to the treatment of the lesion with the riboflavin/UV-A corneal phototherapy.

Despite evidences suggesting the beneficial effect of riboflavin/UV-A corneal phototherapy, different studies report contrasting outcomes [8, 10, 20–23]. Due to this disagreement in the literature, further experimental data are necessary to understand the mechanical behaviour of corneal tissues and represent a prerequisite for the formulation and validation of constitutive models. The latter could be conveniently applied to investigate the mechanical behaviour of corneal tissue in the applied experimental conditions.

To this purpose, uniaxial tensile and stress-relaxation tests were performed in this study to describe the elastic and viscoelastic mechanical behaviour of porcine healthy versus alkali-induced lesioned corneas and to study the effect of riboflavin/UV-A corneal phototherapy.

## Results

### Porcine organ-culture preparation procedure

A total number of 42 cornea samples were tested. All corneas were found to be clear, with no presence of corneal scarring or opacities and transported under sterile condition to the laboratory. The samples were split in different groups: some corneas are analysed in their healthy (H) unaltered state, while other corneas were lesioned (L) by exposing the tissue to an alkaline solution. Part of the samples of each group were analysed in fresh (F) or cultured (C) condition. Corneas from all the groups undergo (Y) or not (N) a cross-linking treatment. All corneas did not develop oedema or infection over the period of cultures. The living corneas in cultures were examined daily using a phase-contrast microscope to monitor the epithelium, the endothelium, and the stromal tissue quality. The porcine organ culture maintained their stromal tissue quality and the cells’ morphology during the 7 days. The swelling of the cornea preserved in the culture medium was weakly present or absent.

### Mechanical testing results

**Fig. 1:**
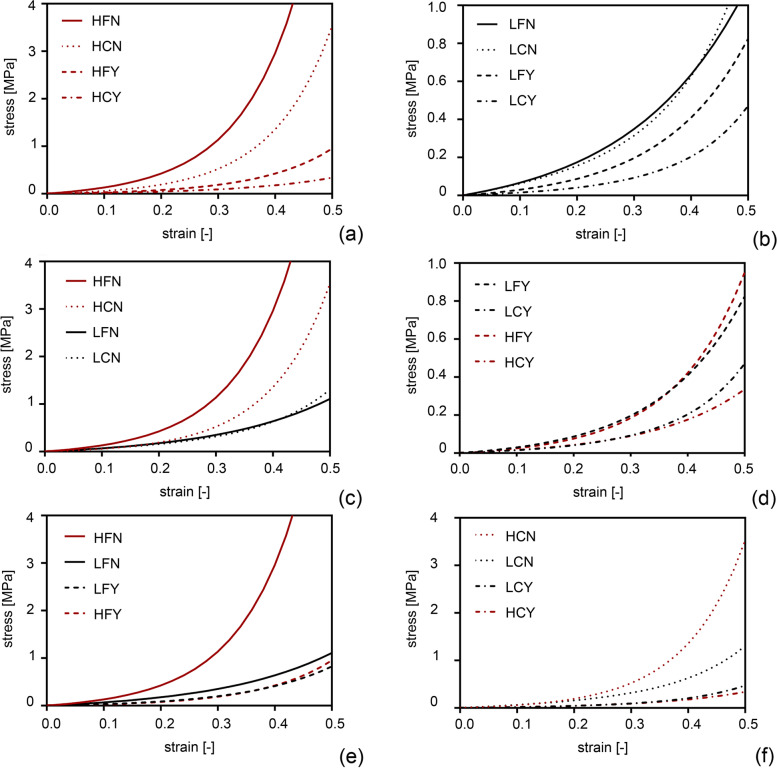
Results from mechanical tests on corneas samples: equilibrium stress-strain data of healthy (H, red lines) (**a**) and lesioned (L, black lines) (**b**) groups, of non-treated (N, continuous and dot lines) (**c**) and treated (Y, dashed and dashed-dot lines) (**d**) groups, of fresh (F, continuous and dashed lines) (**e**) and cultured (C, dashed-dot and dot lines) (**f**) groups. Mean data are reported for each group; data with associated standard deviation (±SD) are available in Supplementary Materials

**Fig. 2:**
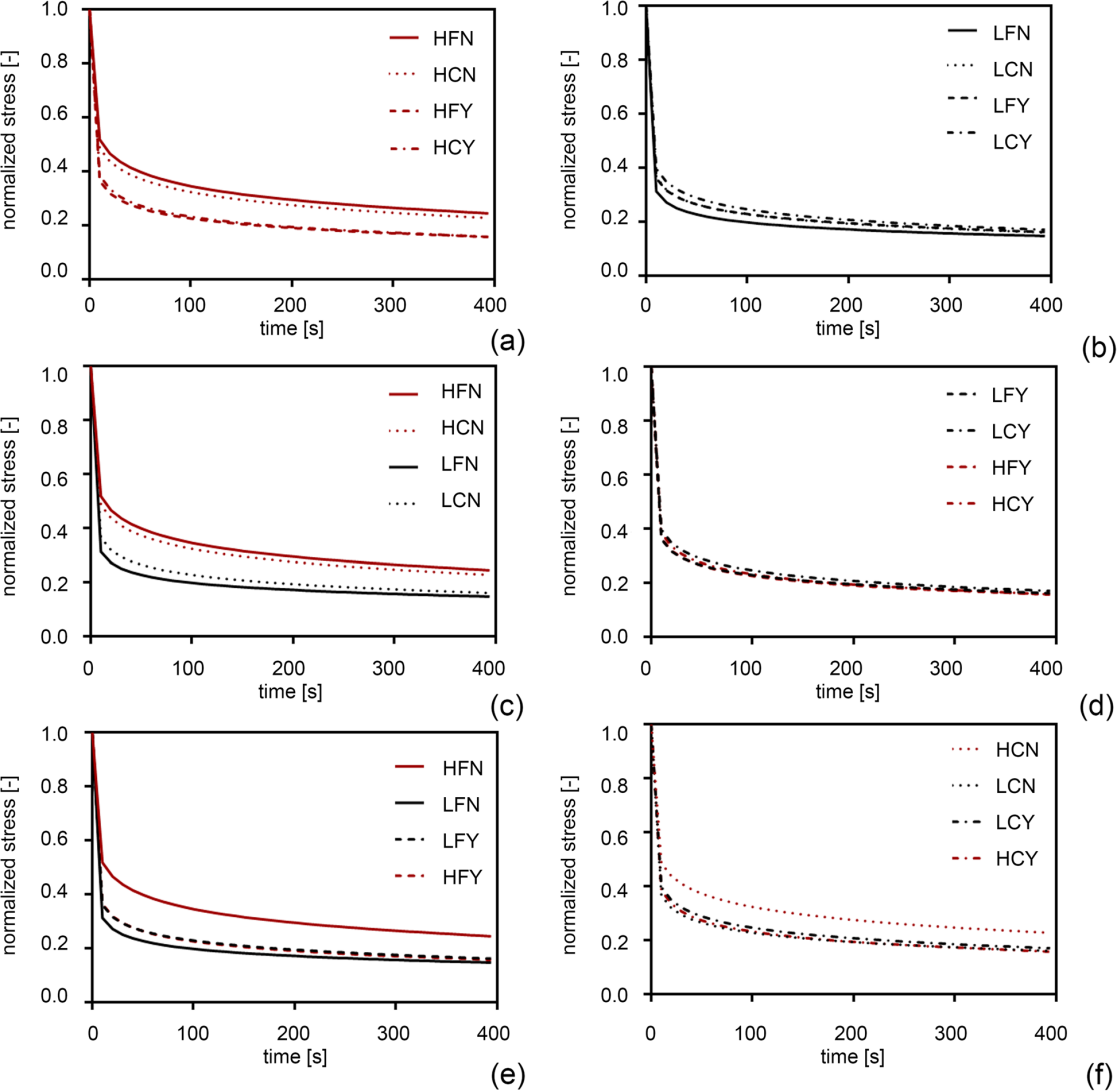
Results from mechanical tests on corneas samples: normalized stress-time data of healthy (H, red lines) (**a**) and lesioned (L, black lines) (**b**) groups, of non-treated (N, continuous and dot lines) (**c**) and treated (Y, dashed and dashed-dot lines) (**d**) groups, of fresh (F, continuous and dashed lines) (**e**) and cultured (C, dashed-dot and dot lines) (**f**) groups. Mean data are reported for each group; data with associated standard deviation (±SD) are available in Supplementary Materials

Experimental results showed the nonlinear and time-dependent behaviour of the cornea. The equilibrium stress-strain response (Fig. 1) is reported for each group considering the mean curves. L group was characterized by lower stiffness, considering the stress-strain almost equilibrium response. At the same time, the Y group showed a decreasing of stiffness both for H and L groups. The normalized stress-time (Fig. 2) behaviour is reported for each group considering the mean curves. Non-treated H groups show higher relaxation times respect to other groups.

### Statistical analysis results

#### Descriptive statistical analysis

With the aim of comparing the mechanical response in H versus L corneas, the mean and standard deviation values were calculated for the output stress and for the output force in the eight corneal populations, from tensile and stress-relaxation data respectively. By descriptive analysis, the relationship between the values of stress vs strain and the values of normalized stress vs time were showed for the eight corneal populations and the results reported in Figs. 1 and 2 were visualized by scatterplot graphs.

#### Inferential statistical analysis

**Fig. 3:**
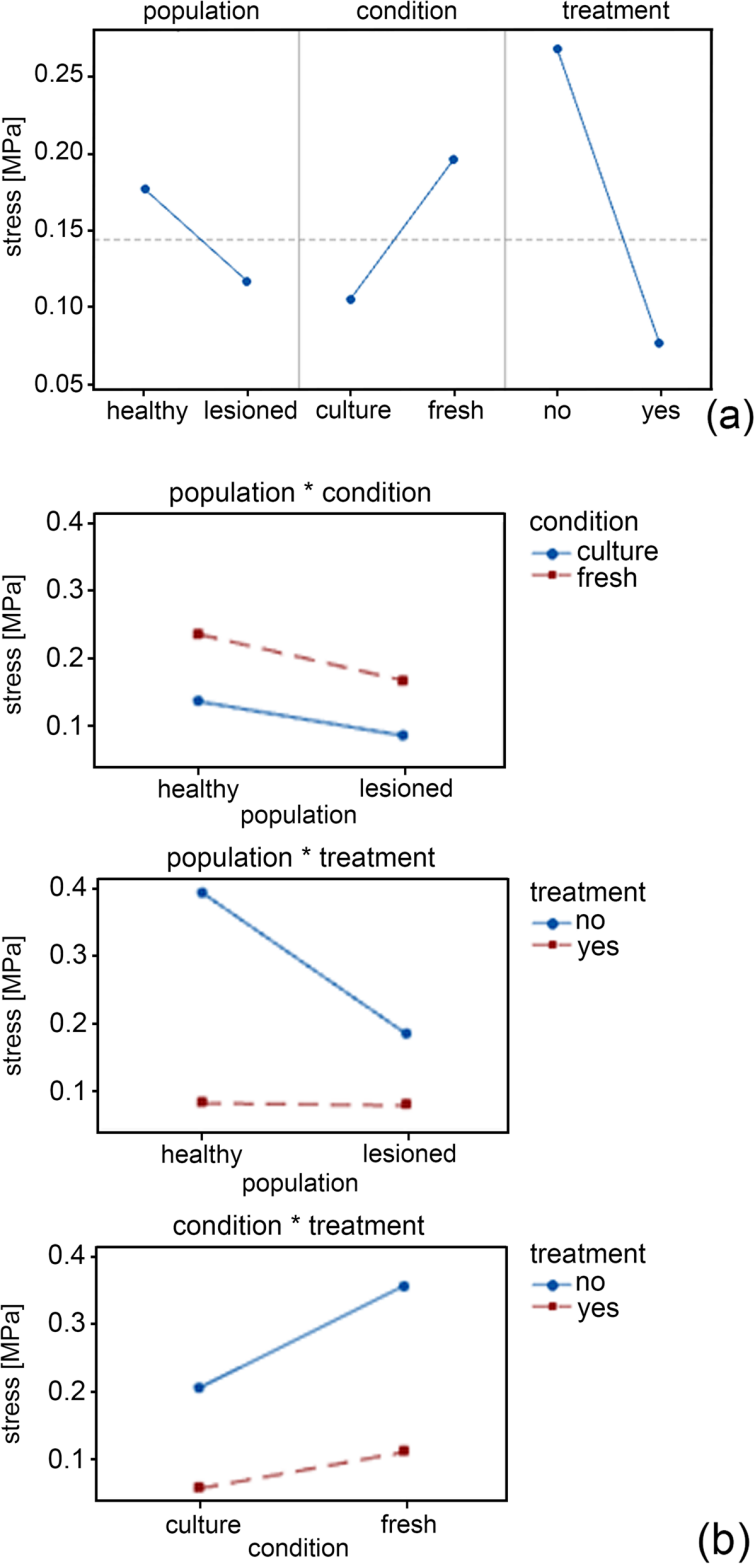
Main effects plot representing the significant results of Table 1 for the output stress in tensile tests (**a**). Interaction plot for the output stress representing the significant results of Table 1 (**b**)

**Fig. 4:**
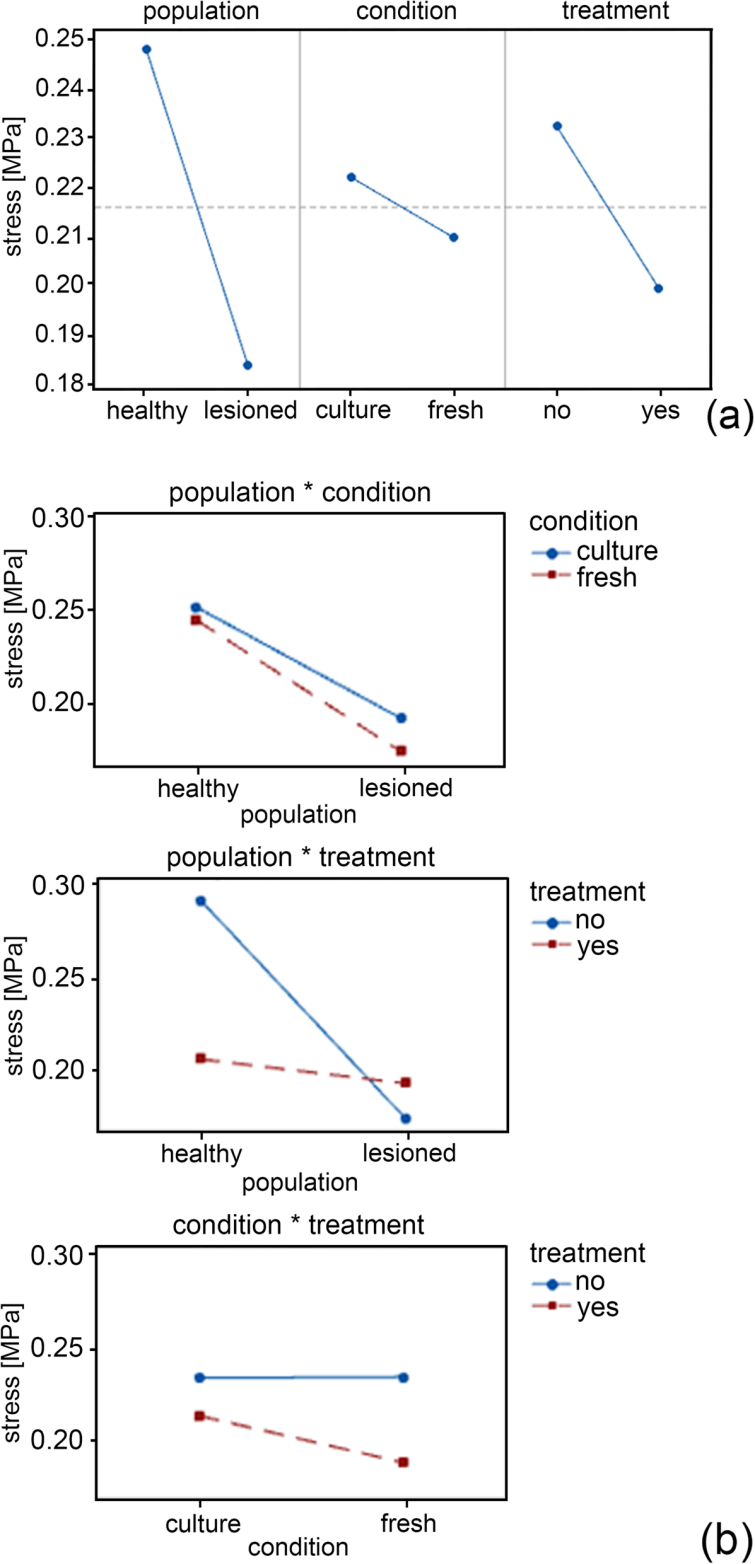
Main effects plot representing the significant results of Table 2 for the output stress relaxation (**a**). Interaction plot for the output force representing the significant results of Table 2 (**b**)

By inferential analysis, a highly significant variation among the factors (population, condition and treatment) was highlighted by a three-way ANOVA between pairs of factors (H vs L, C vs. F and N vs. Y) (Tables 1 and 2; Figs. 3 and 4).

**Table 1:** *P*-value separately for the cofactor strain, the factors (population, condition, treatment); the interaction between cofactor and factors (strain · Population, strain · Condition and strain · Treatment) and the interaction between factors (Population · Condition, Population ·Treatment, Condition · Treatment)

Term	F-value	***P***-value
Strain	2002.79	< 0.001
Strain^2^	338.05	< 0.001
Population	3.67	0.055
Condition	177.97	< 0.001
Treatment	409.47	< 0.001
Strain × Population	36.45	< 0.001
Strain × Condition	8.74	0.003
Strain × Treatment	3.55	0.060
Population × Condition	4.75	0.029
Population × Treatment	164.73	< 0.001
Condition × Treatment	4.43	0.035

**Table 2:** *P*-value separately for the cofactor time, the factors (population, condition, treatment); the interaction between cofactor and factors (time · Population, time · Condition and time · Treatment) and the interaction between factors (Population · Condition, Population ·Treatment, Condition · Treatment)

Term	F-value	***P***-value
Time	64,232.34	< 0.001
Time^2^	28,344.18	< 0.001
Population	21,029.34	< 0.001
Condition	1072.05	< 0.001
Treatment	4075.4	< 0.001
Time × Population	1650.4	< 0.001
Time × Condition	193.31	< 0.001
Time × Treatment	75.55	< 0.001
Population × Condition	329	< 0.001
Population × Treatment	30,019.04	< 0.001
Condition × Treatment	1870.46	< 0.001

A weak variation in the output stress is found between the H and L corneal populations and the p-value is not significant (*p* = 0.055), as shown in Table 1 for the population term. The H population has higher stiffness than L corneal population, as highlighted in Fig. 1. The treatment determines a decrease of stiffness, in fact there is a strong interaction between populations-treatment (*p* < 0.001).

Concerning the results of stress-strain response, main effects plot and interaction are presented in Fig. 3. As shown in Fig. 3a, the stress decreases from H vs L for the population factor, it increases from C vs F for the condition factor and decreases from N vs Y for the treatment factor. In Fig. 3b, all the interaction (population vs condition, population vs treatment, condition vs treatment) are significant, with the interaction population vs treatment showing the strongest variation. These results highlight that there are significant differences in cornea stiffness, as can be also evaluated in Fig. 1: a higher stiffness is found when the treatment is not present and the F condition shows higher stiffness than the C condition. Furthermore, the population-condition interaction is significant (*p* = 0.029), as reported in Table 1.

Concerning the results of stress relaxation behaviour, main effects plot and interaction are reported in Fig. 4. In Fig. 4a, the force decreases from H vs L for the population factor, it decreases from C vs F for the condition factor and from N vs Y for the treatment. As shown in Fig. 4b, all the interaction (population vs condition, population vs treatment, condition vs treatment) are significant, with the interaction population vs treatment presenting the strongest variation.

The interaction plot (Fig. 4) shows that considering the F or C condition, the F condition is showing more variability in stress values and these values are higher in the H population. On the stress parameter, the H population has greater stress values than L population. The F or C condition do not significantly affect stress, and the absence of treatment gives higher stress values. The stress relaxation behaviour of the tissues shows higher relaxation times for non-treated healthy groups (*p* < 0.001) while it is independent from both L and Y groups. On the normalized stress-time curves, the H population has greater values than L population. The F or C conditions do not significantly affect the stress relaxation, and the absence of treatment gives lower relaxation times.

## Discussion

This study is part of an extended research activity aimed at investigating the response of porcine corneas to riboflavin/UV-A phototherapy in the healing of lesions. In a previous study, the structural changes of corneal tissue after UV crosslinking were examined [24]. In detail, the response of porcine cornea to riboflavin/UV-A phototherapy was investigated to get an insight on the injury healing. Riboflavin/UV-A crosslinking treatment showed a significant effect in restoring the damaged stromal structure, recovering the corneal thickness to the values of healthy corneas. In this background, this study is aimed at obtaining new insights also on the effect of riboflavin/UV-A phototherapy on the mechanical properties of corneal stroma. For this purpose, stress-strain and time-dependent mechanical behaviour of cornea are analysed considering the different conditions (treatment, culture and lesion).

Our results indicate that the structural modifications which corneas undergoes in the L and in the T populations influence the mechanical response of the corneal tissue, reducing its stiffness and modifying time-dependent mechanical behaviour. Moreover, the mean values of stress vs. strain highlight a higher stiffness in the case of HFN specimens with respect to other groups. The stress relaxation behaviour shows higher relaxation times for HFN and HCN groups, while it is independent from both L and Y groups. The F or C conditions do not significantly affect the stress relaxation.

These findings would suggest that different micro-structural rearrangements characterize the HFN and HCN corneas. This could be explained by the structural modification that corneas undergo during lesion or treatment that can determine collagen fibres disruption, also modifying their rearrangement over time and the subsequent viscoelastic phenomena [25].

After epithelial and stromal injury, a complex sequence of processes contributes to wound repair and regeneration of normal corneal structure and function [26]. In general, corneal properties are determined by the interaction of collagen, polyanionic ground substance and cells, and the corneal mechanical properties should be considered in relation to corneal structure alteration. The mechanical response of the cornea to injury is dominated by the stroma and only Bowman’s layer and the stroma contain collagen fibrils. The Bowman’s layer consists of an acellular condensation of stroma with more randomly-oriented collagen fibril lamellae [27]. The stromal keratocyte apoptosis has been well-characterized as an early initiating event of the corneal wound healing response. Cornea keratocytes are dormant fibroblast that come into action after injury, playing an important role in healing wounds, synthesizing corneal components. Wilson et al. [28, 29] demonstrated that stromal keratocytes underlying the site of injury undergo apoptosis and, in the degenerative corneal disorders such as keratoconus, apoptosis may contribute to the pathological process. Since cornea properties are determined by the interaction of collagen and cell viability [30], it could be important to consider the corneal mechanical properties in relation to corneal structure alteration. The reduction of cells number in the stroma could determine a decrease in collagen content that causes of a negative effect on the stiffness.

Moreover, it is now well recognized that cells can respond not only to chemical signals, but also to mechanical signals [31]. Lo et al. [32] demonstrated as cell movement is guided by the stiffness of the substrate. An interesting study performed by Molladavoodi et al. [33] demonstrated that corneal epithelial cells are sensitive to changes in substrate stiffness and able to response to substrate stiffness. In detail, they showed as cells displayed a statistically significant lower migration speed on compliant substrates when compared with the stiffer substrates. In this way, corneal epithelial cells respond to changes in substrate stiffness, which may have implications in the understanding and perhaps treatment of corneal diseases, such as keratoconus.

In order to evaluate cell viability in healthy, lesioned and treated corneas, we assessed the number and distribution of cell nuclei in the stromal thickness through image analysis, after histological staining by Haematoxylin and Eosin (H&E) [24]. The cells nuclei of the healthy corneas showed a homogeneous distribution in the stromal thickness. In the damaged corneas, the number of cells decreased in the central part of the cornea, especially in the anterior half of the cornea (loss of cell nuclei in the order of 20–30%). In the riboflavin/UV-A corneal phototherapy treated corneas, the number of cell nuclei did not show an increase after 7 days in culture if compared to the damaged corneas.

Several studies in the literature investigated the effects of UV crosslinking on cornea mechanical properties. Differently from our results, Wollensak et al. [10] experimentally demonstrated a significant increase in corneal stiffness after CXL using uniaxial tensile test in ex vivo models: they measured the effect of collagen cross-linking in human corneas and compare it to the effect in cross-linked porcine corneas having comparable biomechanical properties. They found a significant increase in the stiffness of human corneas by a factor of 4.5 following CXL treatment, while in the porcine samples the increase was of 1.8 only. This increment in the stiffness after collagen cross-linking is caused by the development of intrafibrillar cross-links which are responsible for the increase in fibres diameter. Human corneas showed a stronger increase in the biomechanical stiffness after CXL treatment than porcine corneas: this is caused by the larger portion of cross-linking in the thinner human corneas [10].

Moreover, it must be considered that the cornea is also highly heterogeneous in the central to peripheral region and anteriorly to posteriorly. Also, its mechanical role has been a subject of controversy: Seiler et al. [34] demonstrated as Bowman’s layer is the stabilizing element of corneal curvature due to its assumed mechanical stiffness but Wilson et al. [28] suggested that removal of Bowman’s layer does not measurably alter the mechanical properties of the cornea.

Kohlaas et al. [21] compared the different stiffening effect of cross-linking treatment in the depth of cornea using ex vivo porcine models. The study confirms the depth-dependent difference of cross-links in the cornea after the treatment and suggests the exact depth of biomechanical changes in the corneas caused by this treatment. They demonstrate that the treatment leads to a significant stiffening only in the anterior 200 μm of the corneal stroma: this limited effect is due to the absorption behaviour for UVA in the riboflavin-treated corneas. 65–70% of the irradiation is absorbed in the anterior 200 μm and only 20% in the posterior 200 μm [21].

Furthermore, in an ex vivo study on porcine corneas it was demonstrated that the CXL treatment increases the stiffness only for the anterior flaps obtained from the tissue, while it has an insignificant effect on biomechanical properties if applied on posterior flaps from the incised mid-stromal surface [22]. The suggested reason for this effect is the difference in ultrastructure and composition of corneal tissue: anterior lamellae are more interwoven than posterior ones and the links induced by the treatment occur mainly at the surface of collagen fibrils and in the surrounding proteoglycan network. So, the anterior flaps appear to be stiffer than posterior ones [22].

Matteoli et al. [8] developed a simple procedure using inflation test to evaluate the average elastic modulus of cross-linked portion of ex vivo porcine corneas, assuming its value constant along the depth of the specimen. At the end of the study, they found that corneal thickness is reduced up to 35% immediately after CXL. This is due to strong dehydration of specimens during the treatment: the effect was completely reversible when corneal tissue returned to initial values after immersion in a storage medium. They also found that porcine corneas which underwent CXL treatment were stiffer than untreated ones by 42%: the finding confirms the capacity of CXL to modify the internal structure of the tissue by bundling collagen fibres [8].

However, in a recent study on rabbit corneas, the effect of CXL treatment on the mechanical behaviour of corneal tissue was shown to be in contrast with the literature. In fact, Ortillès et al. [23] demonstrated that using in vivo indentation tests there are statistically significant differences on corneal stiffness between samples that were cross-linked and untreated ones. In particular, corneas that underwent the treatment and were tested for the mechanical behaviour after 7 days from the treatment were less stiff than untreated ones. In the immunohistochemical part of the study, they demonstrated that this reduction in the stiffness on the 7th day after the treatment coincided with a significant reduction in the number of keratocytes. However, corneal stromal cell population returned normal on the 56th day after CXL and the response to indentation tests was similar to untreated samples. Furthermore, they confirmed the nonlinear behaviour of corneal tissue in the in vitro uniaxial tensile tests performed in the same study: they also found statistically significant differences in the mechanical behaviour between samples tested 7 and 56 days after the treatment and untreated ones [23].

Looking at the controversial results reported in the literature, the present study can be considered a relevant contribution in the debate on the effects of alkali-induced lesions and riboflavin/UV-A corneal phototherapy on the mechanical behaviour of cornea.

Nonetheless, some limitations of the present investigation should be mentioned. Uniaxial tensile tests involve corneal tissue cutting and this procedure may induce local damages; moreover, cornea samples are obtained from a curved surface which is flattened during testing. Several variables can affect the mechanical properties, such as the specimens’ species origin, age, preservation method, hydration liquid used, testing protocol, presence of the endothelium and epithelium, level of strain [5]. One additional problem is the preconditioning effects due to protocols of tensile testing which can alter the biomechanical properties of the tissue [8]: in particular, the corneal tissue results stiffer during uniaxial strip tests than in inflation tests [7]. Another important characteristic property of corneal tissue is anisotropy [6]: in this study we use only samples cut in the lateromedial direction, so anisotropy is not evident in the results.

## Conclusion

In this work, the effect of riboflavin/UV-A phototherapy on the stress-strain and time-dependent mechanical properties of cornea is investigated, considering different conditions (treatment, culture and lesion). Experimental results indicate that corneas in the L and in the T populations undergo modifications that affect the mechanical response of the tissue, reducing the stiffness and modifying the time-dependent mechanical behaviour. The stress relaxation behaviour shows higher relaxation times for HFN and HCN groups. These results suggest that different micro-structural rearrangements characterize the HFN and HCN corneas.

This study is part of a larger project in which different treatments for melting keratopathies will be analysed using histological and biomechanical approaches, for improving the healing process. Additional in vitro experimental tests, such as inflation tests, will be developed to identify the structural mechanical behaviour of the cornea. Further analyses aimed at evaluating the effects of different therapies will be performed, including also histological analysis to correlate anatomical configuration with mechanical data. Indeed, lesions and applied treatments induce microstructural rearrangement phenomena of both fibrous elements and ground matrix of the cornea, leading to progressive and significant variation of material stiffness with stretch.

## Methods

### Porcine organ-culture preparation procedure

Porcine eyes were obtained from local abattoirs immediately after slaughter according to Italian and European (86/609/EEC) regulations concerning animal welfare during the commercial slaughtering process. Ethical review and approval were waived for this study, because no live animal was sacrificed for this research. Different experimental conditions were reproduced on fresh or cultured tissues to describe the viscoelastic mechanical behaviour of porcine healthy (H) versus alkali-lesioned (L) corneas and to study the effect of riboflavin/UV-A corneal phototherapy on biomechanical behaviour of corneal tissue. H corneas were tested in their unaltered state, while L corneas were obtained by exposing native tissue to an alkaline solution to create a corneal ulcer. Part of the samples of each group were analysed in fresh (F) or cultured (C) condition. F samples were tested few hours after enucleation, while C samples after 7 days in which the tissue stayed immersed and preserved into a specific culture medium for corneal deturgescence (CARRY-C, produced by Alchimia); further details on methods have been reported in a previous study [24]. The treatment was performed both on H and L samples to evaluate if the cross-linking has a distinct effect in the healing of the tissue. Eight corneal populations defined by the combination of the different experimental conditions are listed in Table 3. A randomized assignment was used to form the corneal populations and, as a consequence, to reduce the error rate.

**Table 3:** Summary of the experimental design, including populations, factors and levels

Populations^a^	Factors	Levels^b^	
Healthy-Culture-No (HCN)	Population	Healthy (H)	Lesioned (L)
Healthy-Culture-Yes (HCY)	Condition	Fresh (F)	Culture (C)
Healthy-Fresh-No (HFN)	Treatment	No (N)	Yes (Y)
Healthy-Fresh-Yes (HFY)			
Lesioned-Culture-No (LCN)			
Lesioned-Culture-Yes (LCY)			
Lesioned-Fresh-No (LFN)			
Lesioned-Fresh-Yes (LFY)			

^a^Combination among 3 factors

^b^Within each factor

**Fig. 5:**
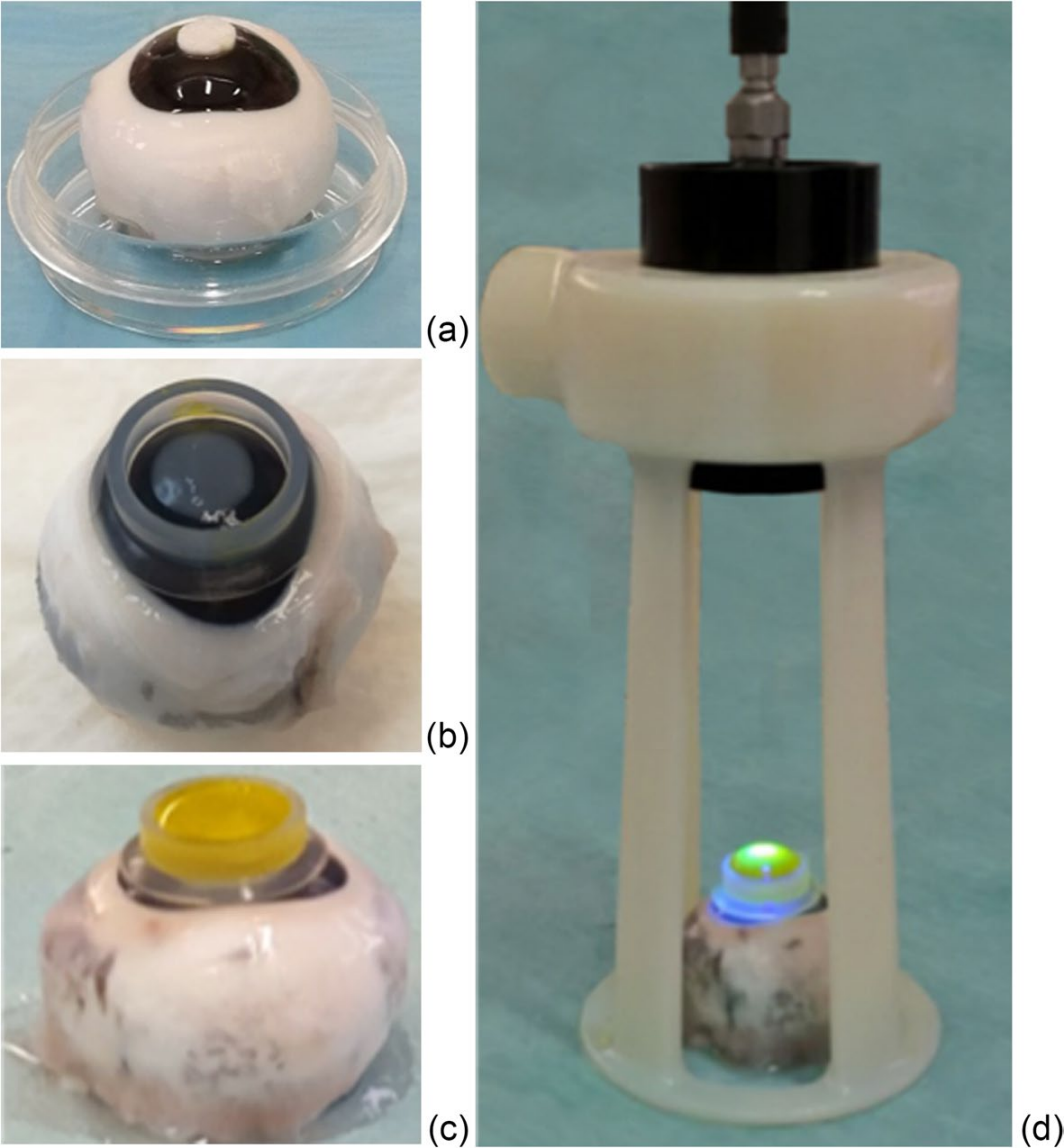
Porcine cornea preparation procedure: porcine eyes showing the filter paper placed on the center of the ocular surface (**a**); application of a plastic ring on the L cornea (**b**); application of isoosmolar 0.1% riboflavin drops into the plastic ring for 30 min (**c**); details of UV-A lamp during riboflavin/UV-A corneal phototherapy (**d**). To obtain the cultured condition, the corneas were isolated from the bulbs and preserved in a specific culture medium for corneal deturgescence (Carry-C^®^ medium, Alchimia, Padua, Italy) for 7 days in an incubator at 37 °C with 5% CO_2_ [24]

The induced lesions on the cornea were produced using a filter paper (0.8 cm) soaked with NaOH (1 N) placed for 1 min on the centre of the ocular surface (Fig. 5a) before wash the corneas with PBS solution for 60 s. The lesion was induced with a chemical technique to obtain an alkali-induced corneal stromal melting like as an experimental model of melting ulcers.

To obtain the treated condition, isoosmolar 0.1% riboflavin drops (Peschke Traid, Huenenberg, Switzerland) were administered into a circular plastic well held firmly against the cornea for 30 min. As suggested by the study of Raiskup et al. [35], the penetration of riboflavin through the epithelium can be increased by different strategies, including increasing contact time. In a previous work [24], we investigated if riboflavin penetrated into the cornea and tested the penetration of riboflavin through the epithelium at different times. Our results showed that the penetration of riboflavin through the epithelium improved to the increasing contact time at 30 min/cornea. All riboflavin stocks were stored in dark and frozen condition prior to any experiment and a new riboflavin syringe was used for each experiment.

The corneas were then UV-A irradiated for 3 min with commercially available equipment (Vetuvir™, Vision Engineering Italy srl, Rome, Italy) with an irradiance was 30 mW/cm^2^ (for total UV-A energy of 5.4 J/cm^2^). UV intensity and wavelength were checked before use. The diameter of irradiation was 9 mm and the UV light was focused on the corneal surface at a distance of 10 cm to optimize the dose of incident light energy (Fig. 5b, c and d).

### Mechanical tests

Mechanical tests were performed at room temperature on rectangular tissue samples obtained from corneal organ culture with a Bose ElectroForce^®^ Planar Biaxial Test Bench instrument (TA Instruments, New Castle, USA), equipped with a load cell of 22 N.

**Fig. 6:**
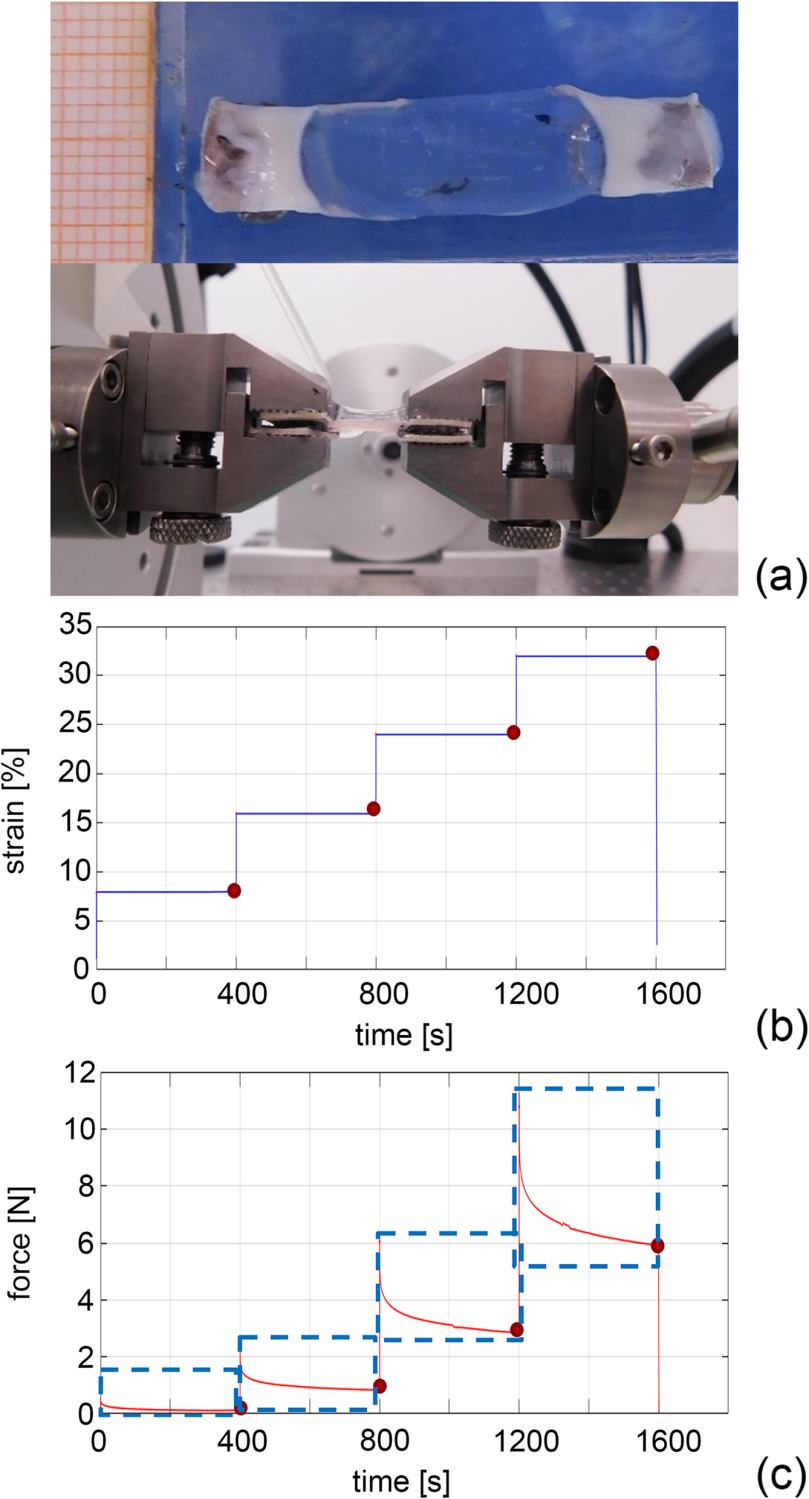
Mechanical tests on corneas rectangular specimens and lateral view of the gripped specimens with glued patches of balsa wood (**a**). Experimental activities based on the assumed strain history (**b**) and the measured force-time data (**c**)

Rectangular samples (width 5 mm × free length 10 mm) were cut from the corneas along medial/lateral direction [6]. The width-to-length aspect ratio was selected in accordance with other test protocols [36, 37]. Sample thickness was measured by means of a digital calliper at different positions through the sample length and an average thickness value was calculated to determine the cross-sectional area of each specimen. Each end of the samples was interposed between two patches of balsa wood to which the Velcro (male side) was glued (Fig. 6a). After that, the samples were clamped by the grips and the closure pressure was adjusted to avoid slippage but, at the same time, damage of the clamped region. The samples were soaked in physiologic saline solution at room temperature prior to testing, and then hydrated by dropping the solution on the sample surface for the entire duration of the test. The number of tested samples for each corneal population is reported in Table 4.

**Table 4:** Number of cornea samples for each population

Populations	N° of samples
Healthy-Culture-No (HCN)	6
Healthy-Culture-Yes (HCY)	5
Healthy-Fresh-No (HFN)	6
Healthy-Fresh-Yes (HFY)	5
Lesioned-Culture-No (LCN)	5
Lesioned-Culture-Yes (LCY)	5
Lesioned-Fresh-No (LFN)	5
Lesioned-Fresh-Yes (LFY)	5

Mechanical tests were carried out following an already validated protocol [38, 39] (Fig. 6b), that includes consecutive loading steps, i.e. stress relaxation at incremental strain values, after tissue preconditioning. The preconditioning was run by applying 10 loading-unloading cycles up to 8% strain, at a strain rate of 1%s^− 1^. Then, the sample was subject to almost-instantaneous elongation (strain rate of 800%s^− 1^) followed by stress relaxation for a time period of 400 s. This procedure was applied to analyse the time-dependent mechanical response, increasing the strain to 8, 16, 24, 32% in consecutive elongation-relaxation steps.

From each test, experimental force-time data, depending on the assumed strain history (Fig. 6c), were post-processed by using a low-pass filtering procedure and a 0.02 N cutting force value was assumed by considering the load-cell sensitivity [40]. Force was then converted to stress, as the ratio between force and initial cross-sectional area of the specimen.

The analysis of the response at the end of relaxation phases led to a curve that shows the equilibrium non-linear response, as the mechanical response when all the time-dependent phenomena completely occur (Fig. 6c). Moreover, stress relaxation data were processed to identify the drop of normalized stress over time, where the normalized stress is defined as the ratio between current stress and peak stress of each rest phase (Fig. 6c). The normalization of relaxation data provides information about the time-dependent behaviour. Equilibrium stress-strain and normalized stress-time curves from each sample were fitted by the following exponential formulations [38, 39], aiming to provide continuous sets of data and to compare results at the same strain/time conditions:

$$\sigma \left(\varepsilon \right)=\frac{C}{\alpha}\left[{\exp}\left(\alpha \varepsilon \right)-1\right]$$

$$\overline{\sigma}(t)=1-{\gamma}_1\left[1-{\exp}\left(-\frac{t}{\tau_1}\right)\right]-{\gamma}_2\left[1-{\exp}\left(-\frac{t}{\tau_2}\right)\right]$$

where *σ* is the equilibrium nominal stress and $\overline{\sigma}$ is the normalized stress (as the ratio between stress at time t and the peak stress value), ε the strain (as the ratio between the elongation and the original distance between grips) and *t* the current time. *c, α* are parameters that are adjusted to fit the non-linear elastic behaviour, while *γ*_1_, *γ*_2_, *τ*_1_, *τ*_2_ are parameters related to the viscoelastic response of the tissue [39].

### Statistical analysis

#### Statistical design

The purpose of this analysis was to investigate the mechanical response in H versus L porcine corneas. The focus of data analytics was to study the significant variations on stress and normalized stress in the eight corneal populations defined by the combination of the two levels of the three factors (population, condition, treatment). In addition, the numerical cofactors (strain for stress and time for normalized stress) were considered as well (Table 3).

#### Statistical analysis: three-way ANOVA and interaction analysis

In order to prove possible significant effects, due to factors and their two-way interaction a three-way ANOVA [41] was performed by using the statistical software Minitab 20 (Minitab^®^ version 19.2020.1 (64-bit), 2020). We applied the one-way analysis for the factors (population, condition, treatment) and Tukey methods with two-way ANOVA for the interaction effect (population – condition; population – treatment; condition – treatment). The analysis of the interaction offers the advantage of highlighting how the combinations of main factors can amplify (or go in the opposite direction) the main effects studied. A *p*-value of less than 0.05 was considered a significant difference. Separately for the two parameters, i.e. Y = (stress [MPa], stress normalized [−]) and X = (strain [−], time [s]), has been formalized the following statistical linear model:

$${\mathrm{Y}}_{\mathrm{i}\mathrm{j}\mathrm{k}}=\mu +{\mathrm{X}}_{\mathrm{i}\mathrm{j}\mathrm{k}}+{{\mathrm{X}}^2}_{\mathrm{i}\mathrm{j}\mathrm{k}}+{\tau}_{\mathrm{i}}+{\beta}_{\mathrm{j}}+{\gamma}_{\mathrm{l}}+{\mathrm{X}}_{\mathrm{i}\mathrm{j}\mathrm{k}}\times \kern0.5em {\tau}_{\mathrm{i}}+{\mathrm{X}}_{\mathrm{i}\mathrm{j}\mathrm{k}}\times {\beta}_{\mathrm{j}}+{\mathrm{X}}_{\mathrm{i}\mathrm{j}\mathrm{k}}\times {\gamma}_{\mathrm{l}}+{\left(\tau\ \beta \right)}_{\mathrm{i}\mathrm{j}}+{\left(\tau\ \gamma \right)}_{\mathrm{i}\mathrm{l}}+{\left(\beta\ \gamma \right)}_{\mathrm{j}\mathrm{l}}+{\varepsilon}_{\mathrm{i}\mathrm{j}\mathrm{l}\mathrm{k}}$$

where τ represents the population τ_i_ = (H - L), β represents the condition β_j_ = (C - F) and γ represents the treatment γ_l_ = (N - Y); the interactions (τ β), (τ γ) and (β γ) represent the three interactions. Finally, we assumed the parameter ε as a normal distribution.

## Supplementary Information

**Additional file 1: Figure S1.** (TIFF) Results from mechanical tests on corneas samples: equilibrium stress-strain data of healthy (H, red lines) (a) and lesioned (L, black lines) (b) groups, of non-treated (N, continuous and dot lines) (c) and treated (Y, dashed and dashed-dot lines) (d) groups, of fresh (F, continuous and dashed lines) (e) and cultured (C, dashed-dot and dot lines) (f) groups. Mean data with associated standard deviation (±SD).

**Additional file 2: Figure S2.** (TIFF) Results from mechanical tests on corneas samples: normalized stress-time data of healthy (H, red lines) (a) and lesioned (L, black lines) (b) groups, of non-treated (N, continuous and dot lines) (c) and treated (Y, dashed and dashed-dot lines) (d) groups, of fresh (F, continuous and dashed lines) (e) cultured (C, dashed-dot and dot lines) (f) groups. Mean data with associated standard deviation (±SD).

## Data Availability

The datasets analysed during the current study are available from the corresponding author on reasonable request.

## Abbreviations

C: Cultured condition
F: Fresh condition
H: Healthy
L: Alkali-lesioned
N: No treatment
Y: Treatment yes
