# Supplementary figures and images for: Mechanical behaviour of healthy versus alkali-lesioned corneas by a porcine organ culture model
Source: BMC Vet Res. 2021 Oct 28;17:340. doi: 10.1186/s12917-021-03050-1 (PMC8555156; doi:10.1186/s12917-021-03050-1)

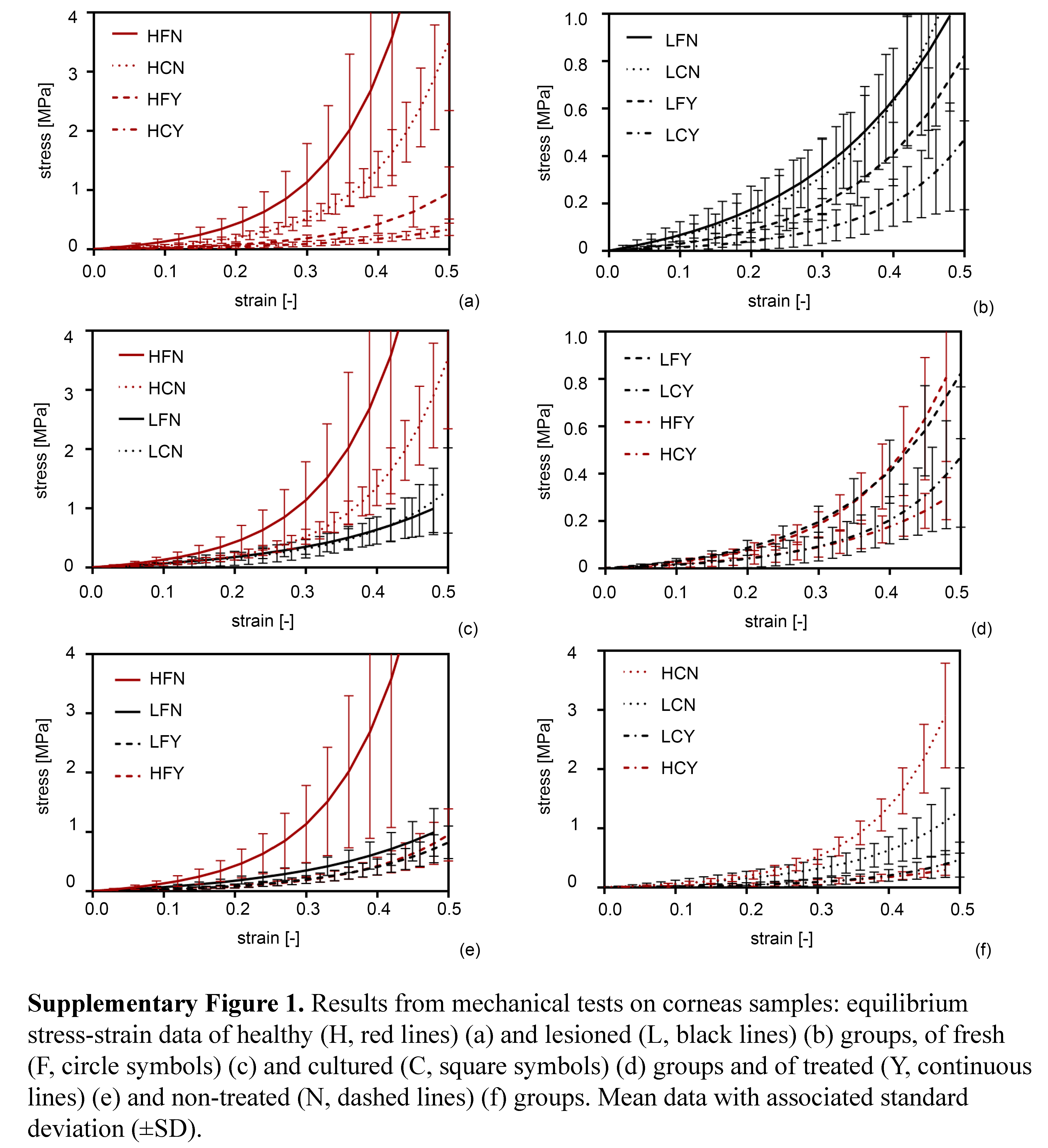

Supplement: Supplementary file 1 — Additional file 1: Figure S1. (TIFF) Results from mechanical tests on corneas samples: equilibrium stress-strain data of healthy (H, red lines) (a) and lesioned (L, black lines) (b) groups, of non-treated (N, continuous and dot lines) (c) and treated (Y, dashed and dashed-dot lines) (d) groups, of fresh (F, continuous and dashed lines) (e) and cultured (C, dashed-dot and dot lines) (f) groups. Mean data with associated standard deviation (±SD). [file 12917_2021_3050_MOESM1_ESM.tif]

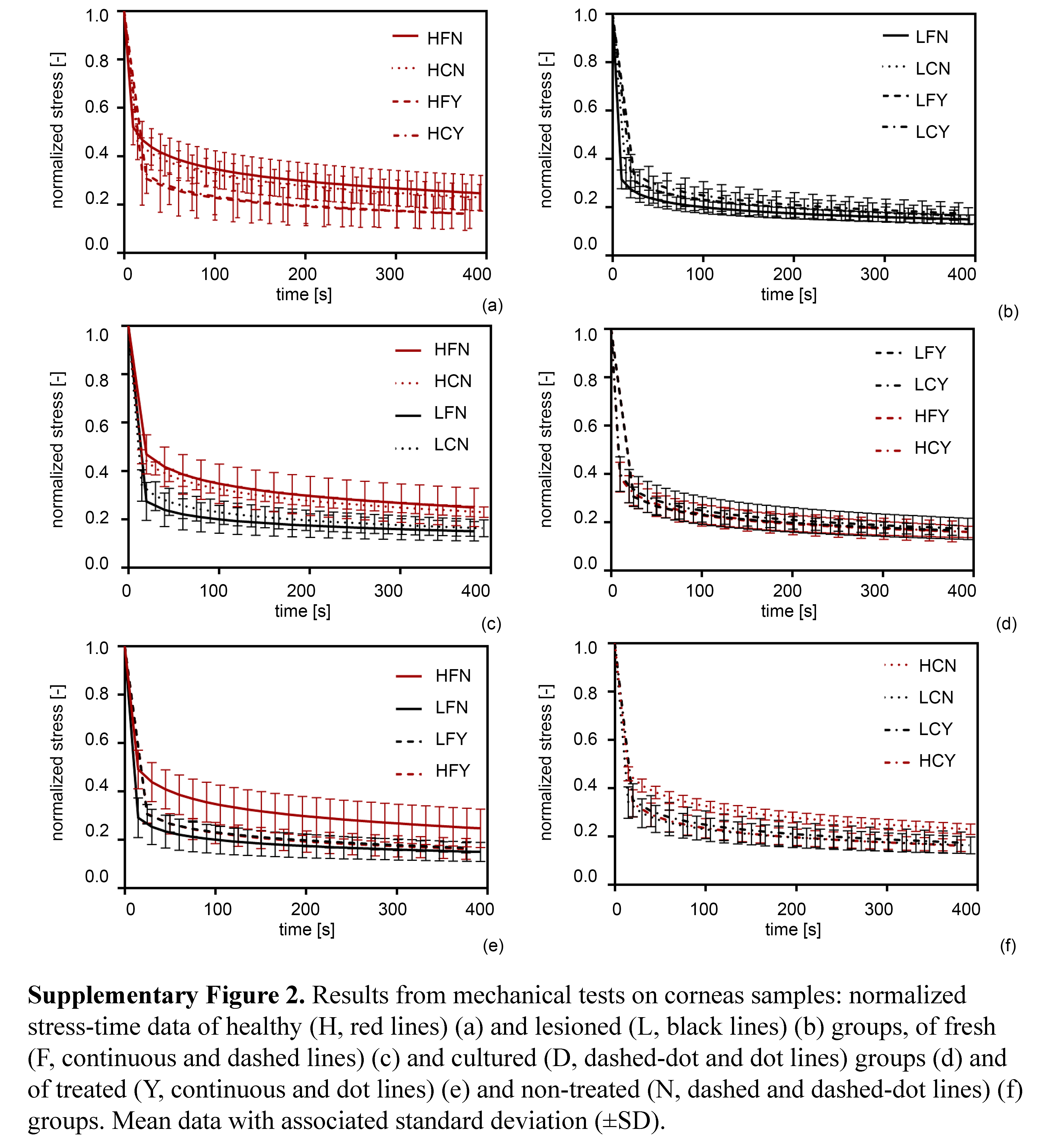

Supplement: Supplementary file 2 — Additional file 2: Figure S2. (TIFF) Results from mechanical tests on corneas samples: normalized stress-time data of healthy (H, red lines) (a) and lesioned (L, black lines) (b) groups, of non-treated (N, continuous and dot lines) (c) and treated (Y, dashed and dashed-dot lines) (d) groups, of fresh (F, continuous and dashed lines) (e) cultured (C, dashed-dot and dot lines) (f) groups. Mean data with associated standard deviation (±SD). [file 12917_2021_3050_MOESM2_ESM.tif]
